# Correction to *Lancet Neurol* 2022; 21: 329–41

**DOI:** 10.1016/S1474-4422(22)00140-5

**Published:** 2022-05

*Morenas-Rodríguez E, Li Y, Nuscher B, et al. Soluble TREM2 in CSF and its association with other biomarkers and cognition in autosomal-dominant Alzheimer's disease: a longitudinal observational study.* Lancet Neurol *2022; **21:** 329–41*—In this Article, the Copyright should read “© 2022 The Author(s). Published by Elsevier Ltd. This is an Open Access article under the CC BY-NC-ND 4.0 license”. In Figure 5, the y-axis of panel 5E should be “Rate of cognitive change (z-score per year)” and the x-axis under panel 5F should be “Estimated years to symptom onset”. These corrections have been made to the online version as of April 13, 2022.

